# Upholding the fundamental values of infection prevention and antimicrobial stewardship in the current political climate

**DOI:** 10.1017/ash.2025.67

**Published:** 2025-04-23

**Authors:** Priya Nori, Gonzalo Bearman

**Affiliations:** 1 Division of Infectious Diseases, Department of Medicine, Montefiore Health System, Albert Einstein College of Medicine, Bronx, NY, USA; 2 Division of Infectious Diseases, Virginia Commonwealth University Health, Richmond, VA, USA

## Abstract

The healthcare and public health communities must adjust to a series of damaging, anti-science, and anti-innovation policies of the new administration. After a brief review of new healthcare and public health-oriented federal policies, we outline priority areas for the infection prevention and antimicrobial stewardship workforce and offer solutions for adaptation.

*“You may not control all the events that happen to you, but you can decide not to be reduced by them.”*

## Maya Angelou

The initial weeks of the new administration heralded an unprecedented attack on American science and innovation through radical actions that appear to conflict with specific legal statutes, violate the constitution, and/or have been temporarily blocked by a federal judge.^1^ Actions specifically targeting healthcare include: 1) withdrawal from the World Health Organization, endangering pandemic preparedness, antimicrobial resistance surveillance, and the elimination of tropical and vaccine-preventable diseases, 2) a freeze of foreign aid through United States Agency for International Development, setting back decades of progress in HIV, malaria, and tuberculosis (TB) control, 3) hostile ultimatums and mass dismissals at the Centers for Disease Control and Prevention (CDC), National Institutes of Health (NIH), Food and Drug Administration (FDA), etc. ensuring a critical loss of expertise in scientific discovery, food and pharmaceutical safety, epidemiology, and outbreak investigation^2^ 5) direct targeting of research and funding for LGBTQ healthcare, 6) censoring of (CDC) websites for references to “gender ideology,” 7) threatening funding to hospitals offering gender transition treatment for individuals under age 19, 8) appointment of a dangerously unqualified and vocal anti-vaccine advocate for Health and Humans Services (HHS) Director as a measles outbreak accelerates in west Texas and New Mexico, marked by the first measles-related death in the United States in 10 years.^3^ 9) an immediate erosion of pandemic preparedness in the shadow of highly pathogenic avian influenza, and 10) a marked decrease in indirect research funding from the NIH. These measures greatly erode the U.S.’s long-standing position as a science, technology, health security, and innovation leader.^4^ Globally, decades of progress and potentially millions of lives will be lost or worsened through direct and indirect threats to healthcare and public health.

Locally, outcomes related to maternal-fetal health, sepsis, healthcare-associated infections, and antimicrobial resistance, may worsen as the public health workforce coordinating surveillance, treatment guidelines, and outbreak mitigation efforts is threatened. Additionally, academic institutions and hospitals that receive the most in (NIH) funding stand to lose more than $100 million annually on average, impacting potentially thousands of healthcare jobs.^5^ Other vulnerable programs may include post-graduate training programs, federally qualifying health centers, Ryan White clinics, the Vaccines for Children program, and multiple others.

How do these regressive policies impact the infection prevention or antimicrobial stewardship workforce? What remains under our influence and how should we adapt (Table 1)?

**Table 1.** What is at stake and how do we adapt?

Domain	What is at stake	Adaptation
Patient care	Funding cuts to crucial programs for marginalized patient groups, patients may fear retribution or exposure while accessing healthcare	• Harness telehealth services, collaborate with philanthropic or non-governmental organizations to support funding gaps • Harness trusted relationships to reinforce vaccine knowledge and uptake
HAI prevention and surveillance	Loss of crucial public health expertise to coordinate surveillance and mitigation efforts	• Rely heavily on SHEA compendia and known HAI prevention strategies • Uphold society publications as a repository for HAI data
Stewardship/AMR surveillance	Loss of public health expertise, gaps in scientific/epidemiologic investigation and publication on MDROs like *C.auris*, CRAB, etc. Loss of coordination with WHO	• Continue to submit, review, disseminate NHSN AUR data to inform local stewardship • Partner with external groups for global AMR surveillance data^6^
Pandemic preparedness	Loss of expertise and coordination of multiple federal agencies (eg, CDC, HHS, USDA, etc.), vulnerable supply chains, loss of coordinated federal alerts, guidance from advisory committees	• Rely on local expertise to fill in gaps, professional society journals and websites can serve as a repository of crucial information
Advocacy and activism	Appointment of unqualified leaders, promotion of regressive anti-science and harmful policies, direct patient harm, and lack of coordinated opposition	• Actively participate in professional society advocacy opportunities at a local and federal level • Join mission-oriented advocacy groups^7^ • Support issues important to you like climate health, health equity, etc.

## Be clear on our immediate responsibility to patients

As frontline healthcare workers (HCW), we remain principally committed to the well-being of our patients. Despite a rising anti-science and health-freedom movement, the public’s trust in personal physicians is greater than that of the healthcare system.^6^ At the local level, we must advocate for ongoing access to (HIV and STI) testing and treatment and prevention services. We must continue to cultivate trusted provider-patient relationships to ensure the dissemination of accurate vaccine information and uptake. We should leverage telemedicine services to maximize contact with trusted providers, especially when at-risk patients (ie, those from marginalized or undocumented groups) feel unsafe leaving their homes. Health systems must prepare for an influx of patients, visitors, and staff with vaccine-preventable diseases. Infection preventionists should guide peer education and trust-building to prevent further erosion in (HCW) vaccine uptake due to a potential retraction of local vaccine mandates.

## Hold steadfast to the principles of healthcare-associated infection (HAI) prevention, surveillance, pandemic preparedness

Rapid changes at the (CDC and DHHS) require infection prevention and stewardship teams to understand which surveillance data is presently available through the National Healthcare Safety Network (NHSN), and access and disseminate it regularly to inform local efforts.^7,8^ Other data sources from not-for-profit, privately funded organizations can be useful to fill in certain gaps.^9^ States rely heavily on federal funding to support local public health departments and Medicaid, however, working closely with local public health jurisdictions can enable maintenance of (HAI and AMR) surveillance and communicable disease transmission data, like waste-water surveillance data where available.^10^ Additionally, we must lean heavily on proven interventions, like hand hygiene and (HAI) prevention bundles; peer-reviewed, published guidance like (SHEA) compendia^11^ are enduring and less likely to be censored or removed from websites.

While scientific investigation and innovations are on hold, we must shift our focus to direct patient care and protecting vulnerable patients in a hostile environment. We should ensure ongoing hospital readiness for (HPAI), Mpox, viral hemorrhagic fever, and vaccine-preventable illnesses by updating emergency preparedness exercises and simulations with help from local public health partners and high-consequence pathogen referral centers. Given ongoing supply chain concerns due to strained relations with global partners, we must ensure personal protective equipment (PPE) stockpiles and adjust (PPE) use in times of scarcity, learning from recent experience. Moreover, the suspension of expert advisory groups like the Healthcare Infection Control Practices Advisory Committee, the Presidential Advisory Council on Combating Antibiotic-Resistant Bacteria, and the Advisory Committee on Immunization Practices indicates that we must rely more heavily on professional society guidance and hospital-based expertise.

## Relentlessly pursue antimicrobial stewardship and antimicrobial resistance (AMR) surveillance

While temporarily unavailable to scrutinize language, the (NHSN) Antibiotic Use and Resistance portal remains active as of February 2025. Programs can continue to submit, receive, and report data to inform local stewardship initiatives. Antimicrobial resistance mitigation and funding had bipartisan support, given the success of professional society advocacy efforts and direct patient stories.^12^ Moreover, robust state-level stewardship efforts in states like Minnesota and Tennessee remain a model for hospital–public health partnerships to maximize the impact of (HAI, AMR, and AU) data.^13^ COVID-19 and Mpox cemented stewardship programs as a major leg of pandemic preparedness given expertise in experimental treatment and vaccine allocation and dissemination pathways.^14^ Likewise, hospital-based stewardship programs should ensure adequate stockpiles of antivirals to treat HPAI should there be an influx of admissions.

While new drug development for (AMR) remains inadequate,^15^ federal funding cuts will further impede the United States FDA new anti-infective and vaccine approvals.^16^ However, on February 7, 2025, the (FDA) approved a new monobactam/B-lactamase inhibitor combination to treat complicated intra-abdominal infections, which adds to the armamentarium for extensively drug-resistant Gram-negatives.^17,18^

## Leverage professional societies to fill in vital health information and leadership gaps

The healthcare community is encouraged by professional societies’ clear, steadfast, and renewed commitment to their missions. They are poised to fill the void with expert statements, guidance, and clinical pathways. Professional societies greatly enhanced and consolidated advocacy efforts and collaboration with other medical societies and have been vocal in the media about the negative impact of new policies on patients. Further enhancement can occur through collaboration with non-governmental or philanthropic organizations and the pharmaceutical industry to support at-risk programs, like Ryan White clinics.

Professional societies and their partners can pressure industries directly benefiting from new federal policies to protect the health of their employees, customers, and surrounding communities. Professional societies should continue to publish research on HAIs, stewardship, AMR, and health equity outcomes in society journals and ensure this information is archived and accessed in perpetuity. They should continue close communication with members about emerging infections, pandemic threats, (HAIs, AMR), etc. They should strengthen collaborations and information-sharing with professional organizations outside the U.S. with close ties to the (WHO), like the European Society of Clinical Microbiology and Infectious Diseases. Professional societies should intensify knowledge dissemination on virtual platforms created during the pandemic, like Society for Healthcare Epidemiology of America town halls and the Infectious Diseases Society of America Realtime Learning Network. Finally, professional societies should continue to seek member input and maintain protected spaces to voice concerns in a safe, organized fashion.

## Your voice matters

Healthcare professionals should stay informed via trusted information sources without becoming overwhelmed. They should reinforce accurate health information during clinical encounters and ensure patients understand their rights. They should intensify commitment through professional society advocacy opportunities, letters to legislators, and visits to Capitol Hill. They should stand in solidarity with the public health and research communities gravely impacted by regressive federal policies. They should continue civic engagement in issues they’re passionate about, like climate advocacy, LGBTQ+ rights, and maternal-fetal health.

## Conclusion

These are unprecedented times with a dire erosion of public health and research infrastructure, and threats to crucial federal programs which serve as a lifeline for our patients. To avoid becoming overwhelmed and despondent, we encourage readers to remain focused on what remains under our control and influence. This includes leveraging patient-provider relationships and remaining steadfast in our commitment to patient safety. Active engagement with professional society initiatives is critical for protecting infection prevention and stewardship priorities, maintaining high standards for patient care, advocating for ongoing research funding, and evidence-based public health policies. Challenges and setbacks should be viewed as opportunities to exercise courage and re-commitment to our fundamental values.
